# Eye disorders spectrum: a tertiary hospital pediatric ophthalmology clinic based in Ethiopia

**DOI:** 10.1186/s12886-022-02336-8

**Published:** 2022-03-12

**Authors:** Yohannes Tewolde Kidane, Addisu Worku Teshome

**Affiliations:** Addis Ababa University, College of Health Science, School of Medicine, Department of Ophthalmology, 31531, Addis Ababa, Ethiopia

**Keywords:** Eye disorders, Pediatric eye clinic, Spectrum, Tertiary hospital

## Abstract

**Background:**

Epidemiological studies to determine the pattern of eye disorders among children are important for proper health care planning and management. This study aimed to document the spectrum and frequency of eye diseases in children who attended the pediatric ophthalmology clinic of a tertiary teaching hospital in Addis Ababa, Ethiopia.

**Methods:**

A cross-sectional and convenient sample of 1237 male and female children (16 years and below) with ocular disorders presenting for the first time and those children with a settled diagnosis coming for a follow-up visit between June 1, 2018, and May 31, 2019, were included in the study. Data on presentation age, sex, and diagnosis were collected and analyzed. Eye disorders were classified into various categories. Children were grouped into four age groups. Ratios, percentages, and chi-square associations were calculated. *P* < 0.05 was considered statistically significant.

**Results:**

Of the children, 60% were male. The mean age (standard deviation) of the children was 4.26 (± 4.1) years. Patients aged 0– < 6 years old were the largest group, constituting 70.5%. Ocular motility imbalances were the most common ocular disorders seen (32.8%), followed by childhood cataract (18.4%) and infection and inflammation of the eye and adnexa (8.3%). Ocular motility imbalances were observed more frequently and statistically significantly (*p* < 0.001) in children aged 1- < 6 years. Within the childhood cataract category, congenital cataracts were more prominent (7.1%). Within the infection and inflammation category, corneal/scleral infections were more common (3.7%).

**Conclusions:**

The study highlights common eye disorders seen in children in a specialized hospital ophthalmic clinic. Ocular motility imbalance, childhood cataracts, infection and inflammation of the eye and adnexa were the most commonly occurring disorders. Early presentation was common, and males were more affected than females.

## Introduction

The pattern of pediatric eye disorders varies greatly around the world and is largely determined by socioeconomic development and the availability of primary health care and eye care services [1]. In high-income countries, lesions of the optic nerve and higher visual pathways predominate as the causes of blindness, while corneal scarring from measles, vitamin A deficiency, the use of harmful traditional eye remedies, and swelling of the child’s eyelids due to infection during birth are the major causes of blindness in developing countries. Retinopathy of prematurity is an important cause in middle-income countries [2]. Other significant causes in all countries are congenital abnormalities, such as cataracts, glaucoma, and hereditary retinal dystrophies [1].

The worldwide prevalence of blindness is 0.78/1000, and there are an estimated 1.4 million blind children, three-quarters of whom live in developing countries in Africa and Asia [3]. In Ethiopia, the prevalence of childhood blindness was 0.1%, accounting for 6% of overall blindness [4].

There is a lack of accurate and reliable data on the pattern of pediatric eye disorders at Menelik II Hospital, a tertiary pediatric eye care center. Because prevention and treatment of childhood blindness are disease specific, a description of the pattern of eye disorders in children is essential [1]. There are few hospital-based studies on the pattern of pediatric eye disorders in Ethiopia [5, 6]. Given the variation in study designs, sample sizes, the effects of different geographical and socioeconomic situations and relative importance that can change over time, we wanted to document the pattern of eye disorders in a pediatric population over one year and with greater participant numbers than previously. The findings obtained from this hospital-based study are needed for planning and evaluating preventive and curative services for children to reduce severe visual impairment and blindness.

## Methods

A hospital-based prospective cross-sectional study was conducted at Menelik II Hospital from June 1, 2018, to May 31, 2019. It is a tertiary eye care teaching hospital located in the capital city of Ethiopia and serves as a referral hospital for a number of hospitals in its referral chain from all regions of the country.

During the study period, all consecutive children under 16 years of age with eye disorder presenting for the first time and those children with settled clinical diagnosis coming for follow-up at the hospital pediatric ophthalmology clinic and seen by the consultant pediatric ophthalmologist were included.

Ocular examination was performed starting with visual acuity using different methods for age. Fixation and the ability to follow light in infants, colorful fixation targets and CSM (central, steady and maintained) methods were used to assess visual acuity in 1- to 4-year-old children. For children 4 years and older, Snellen’s visual acuity chart was used. The Hirschberg corneal reflex was used quickly to check ocular alignment. A cover-uncover test was performed to diagnose strabismus and hetrophoria. A slit lamp, pen torch light, and a magnifying loupe were used to evaluate the eyelid margins, conjunctivae, cornea and anterior segment of the eyes for any abnormality. Intraocular pressure measurement was performed using a Perkin handheld tonometer in suspected cases of childhood glaucoma when the child got calm or slept. A direct ophthalmoscope, 90 Diopter Volk, and both were used to examine the posterior segment after dilatation of both pupils using 1% mydriacyl. Cycloplegic refraction was routine. Ptosis evaluation included vertical fissure height, lid margin reflex distance, lid to crease distance, and a levator function test using a ruler. Laboratory investigations and imaging were performed whenever required to elicit a diagnosis. A multidisciplinary consultation was approached with other ophthalmic subspecialists, pediatricians, and other discipline specialists. An examination under anesthesia was carried out to confirm a diagnosis when needed.

An eye disorder that was reported for each patient in the study was one of the main diagnoses that was primarily responsible for the pediatric ophthalmology clinic services. In those children with settled clinical diagnoses coming for follow-up visits during the study period, the initial diagnosis was accepted as a disorder.

Ethical approval and permission to conduct the study were obtained from the ethical review committee of the Department of Ophthalmology, School of Medicine, Addis Ababa University and were carried out in accordance with the tenets of the Declaration of Helsinki. Parents, guardians, or both were informed of the purpose of the study and had to give their oral informed consent before the child was enrolled.

A structured questionnaire was used to collect data from children who attended the pediatric eye clinic. On a daily basis, age at presentation, sex, and clinical diagnosis were extracted.

The disorders were grouped into 14 categories; any disorder that did not fit into these categories was included in other categories. The disorder categories included were ocular motility imbalance, childhood cataract, congenital anomalies, infection/inflammation, orbital/ocular tumors, orbital, ocular and both trauma, childhood glaucoma, refractive error, nasolacrimal duct obstruction, ocular allergies, ptosis, cortical visual impairment/blindness, retinopathy of prematurity, and ectopia lentis.

For age-related eye disorders, patients were grouped by age interval into infants (0-<1 year), toddlers and preschool (1-<6 years), school age (6-<11 years) or older children and teenagers (11-16 years). Data entry on the computer was performed by using an Excel spreadsheet, and data analysis was performed using SPSS version 20 software. A chi-square test was used to compare variables, and a *p* value of less than 0.05 was considered statistically significant. Ratios and percentages were calculated. Possible associations with age groups and sex, age groups and ocular disorders, and sex and ocular disorders were pursued using *p* values.

## Results

**Fig. 1:**
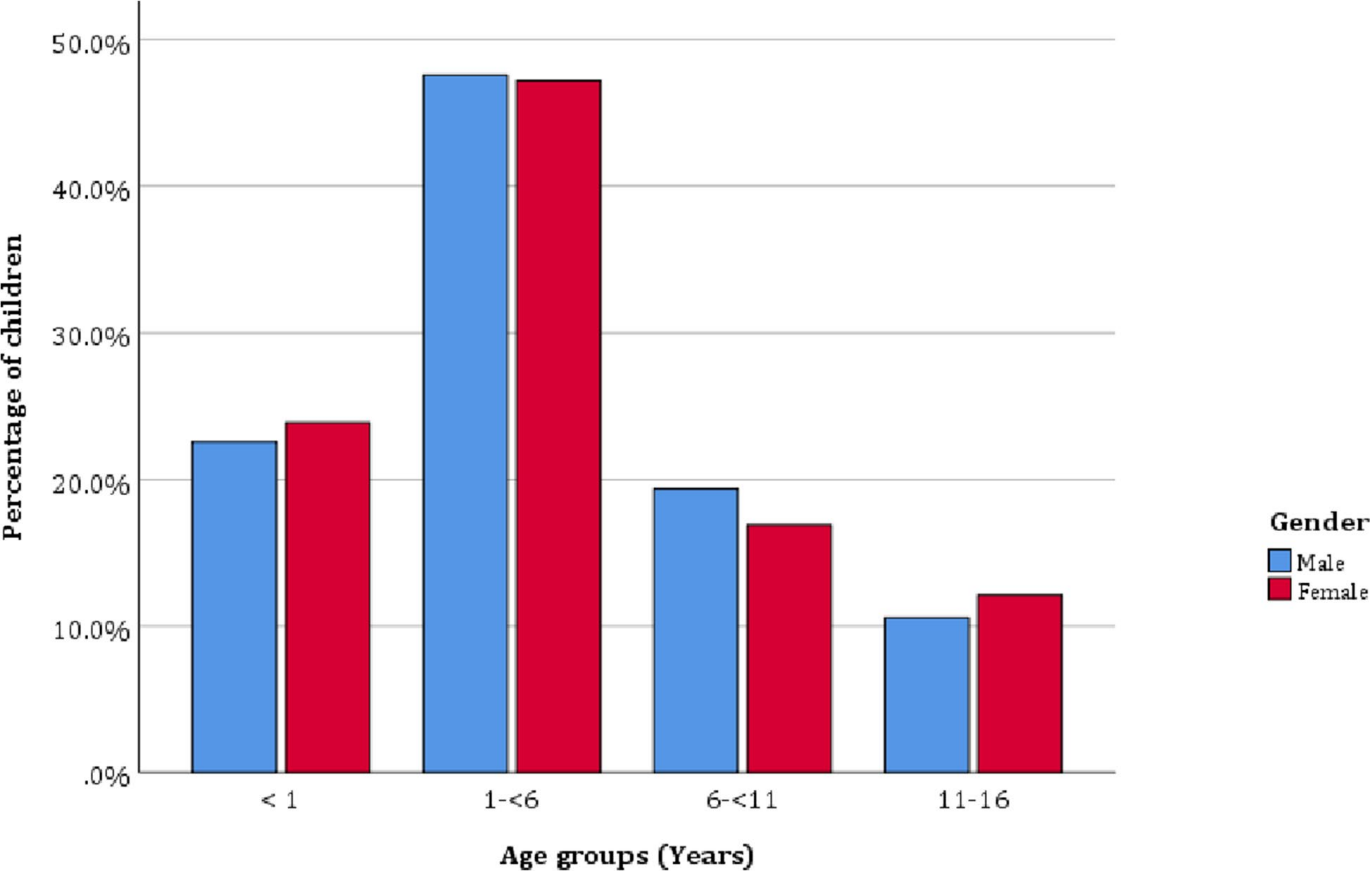
Age group and sex distribution of children seen at the pediatric ophthalmic clinic

A total of 1326 new and follow-up pediatric patients who were seen in the pediatric ophthalmic clinic at Menelik II Hospital during the study period and who met the inclusion criteria were recruited for this study. As incomplete questionnaires were discarded, data obtained from 1237 respondents were analyzed, resulting in a response rate of 93.3%. The mean (± SD) age of the pediatric patients evaluated in the pediatric ophthalmic clinic was 4.3 (± 4.1) years. Their ages ranged from 5 days to 15 years. There were 692 (60%) males and 545 (40%) females. Figure 1 shows the age group and sex distribution of the children seen, with a male preponderance in all age groups. Fig. 1 also shows that the highest percentage of consultation was recorded among pediatric patients up to 6 years of age, constituting 70.5% of the patients (Chi Square, *p* = 0.272), with a maximum number of patients (47.4%) presented between the 1- and <6 -year age groups. There was almost equal sex distribution across all age groups.

Table 1 shows the spectrum and frequency of various pediatric eye disorders seen. Eye motility disorders were the most common disorders seen (32.8%). This was followed by childhood cataract (18.4%), infection/extraspace inflammation (8.3%), orbital/ocular tumors (7.1%) and congenital anomalies (6.8%). The most common form of ocular motility imbalance was convergent strabismus (18.8%).

**Table 1:** Spectrum of pediatric eye disorders as a percentage of the total sample

**Ocular disorders**	**number**	**%**
**Ocular motility imbalance**	**406**	**32.8**
Convergent strabismus	232	18.8
Divergent strabismus	110	8.9
Vertical strabismus	10	0.8
Complex strabismus	19	1.5
Nystagmus	23	1.9
Amblyopia	12	1.0
**Childhood cataract**	**227**	**18.4**
Congenital cataract	88	7.1
Traumatic cataract	76	6.1
Developmental cataract	63	5.1
**Infection/inflammation**	**103**	**8.3**
Lid (Stye/chalazion/blepharitis)	14	1.1
Conjunctiva (viral/bacteria)	17	1.4
Cornea/sclera	46	3.7
Uvea	11	0.9
Vitreous/retina	6	0.5
Optic nerve	9	0.7
**Orbital/ocular tumor**	**88**	**7.1**
Retinoblastoma	66	5.3
Rabdomyosarcoma	10	0.8
Capillary hemangioma	6	0.5
Optic nerve glioma	6	0.5
**Congenital anomalies**	**84**	**6.8**
Congenital entropion/ectropion/epiblepharon	20	1.6
Dermoid cyst	18	1.5
Microphthalmia	14	1.1
Congenital Hereditary Endothelial Dystrophy	5	0.4
Coloboma	11	0.9
Persistent fetal vasculature (PFV)	9	0.7
Retinitis Pigmentosa (RP)	3	0.2
Congenital Cranial nerve palsy (CN III/IV/VI)	6	0.5
**Childhood glaucoma**	**76**	**6.1**
Congenital glaucoma	55	4.4
Juvenile open angle glaucoma	10	0.8
Secondary glaucoma	11	0.9
**Refractive error**	**64**	**5.2**
Myopia	34	2.7
Hyperopia	23	1.9
Astigmatism	7	0.6
**Ocular/orbital trauma**	**52**	**4.2**
Blunt trauma	20	1.6
Penetrating injury	17	1.4
Non penetrating injury	8	0.6
Superficial foreign body (Conjunctiva/cornea)	8	0.6
**Ocular allergies**	**36**	**2.9**
Vernal keratoconjunctivitis	22	1.8
Other allergies	14	1.1
**Congenital Nasolacrimal duct obstruction**	**33**	**2.7**
**Ptosis**	**25**	**2.0**
**Cortical visual impairment/blindness**	**17**	**1.4**
**Retinopathy of prematurity**	**10**	**0.8**
**Ectopia lentis**	**6**	**0.5**
**Others**	**10**	**0.8**
**Total**	**1237**	**100**

Blunt ocular trauma (1.6%) was the most common form of orbital and ocular trauma seen, followed by penetrating injuries (1.4%) and superficial foreign bodies (0.6%). Under the category of others, they included pseudostrabismus (0.16%), conjunctiva nevus (0.48%) and coat disease (0.16%)*.*

The spectrum and frequency of eye diseases varied across the four age groups (Table 2). The difference in presentation by age group was statistically significant among the children with different eye diseases. Ocular motility imbalance was predominant (58.4%) among the 1-<6 year age group (Chi-Square, *p*<0.001). Childhood cataracts were the most prevalent disorder (35.7%) affecting the age group 6-<11 years (Chi-Square) *p*<0.001). The prevalence of congenital anomalies (81.0%) was significantly higher in children under 6 years (chi-square, *p*<0.001).

**Table 2:** Distribution of pediatric eye disorders across age groups among children attending the pediatric eye clinic

**Ocular disorders**	**Age group (years)**				
	** < 1 yr (%)**	**1- < 6yrs (%)**	**6- < 11yrs (%)**	**11-16yrs (%)**	Total (%)
Ocular motility imbalance	49 (12.1)	237 (58.4)	59 (14.5)	61 (15.0)	406 (32.8)
Childhood cataract	48 (21.1)	81 (35.7)	63 (27.8)	35 (15.4)	227 (18.4)
Infection/inflammation	23 (22.3)	50 (48.5)	23 (22.3)	7 (6.8)	103 (8.3)
Orbital/ocular tumor	14 (15.9)	60 (68.2)	8 (9.1)	6 (6.8)	88 (7.1)
Congenital anomalies	42 (50.0)	26 (31.0)	12 (14.3)	4 (4.8)	84 (6.8)
Childhood glaucoma	43 (56.6)	20 (26.3)	9 (11.8)	4 (5.3)	76 (6.1)
Refractive error	7 (10.9)	32 (50.0)	18 (28.1)	7 (10.9)	64 (5.2)
Orbital/ocular trauma	7 (13.5)	22 (42.3)	18 (34.6)	5 (9.6)	52 (4.2)
Ocular allergies	3 (8.3)	11 (30.6)	13 (36.1)	9 (25.0)	36 (2.9)
Congenital nasolacrimal duct obstruction	12 (36.4)	21 (63.6)	-	-	33 (2.7)
Ptosis	10 (40.0%)	14 (56.0)	-	1(4.0)	25 (2.0)
Cortical visual impairment/	14 (82.4)	3 (17.6)	-	-	17 (1.4)
Retinopathy of prematurity	10 (3.5)	-	-	-	10 (0.8)
Ectopia lentis	-	4 (66.7)	2 (33.3)	-	6 (0.5)
Others	4 (40.0)	5 (50.0)	1 (10.0)	-	10 (0.8)
**Total**	**286 (23.1)**	**586 (45.4)**	**226 (18.3)**	**139 (11.2)**	**1237 (100)**

This study showed that there were statistically significant differences in eye disorders in male and female children (chi-square, *p* = 0.015) (Table 3).

**Table 3:** Sex distribution of eye disorders in children attending the pediatric eye clinic

**Ocular disorders**	**Sex**			
	**Male (%)**	**Female (%)**		**Total (%)**
Ocular motility imbalance	222 (54.7)	184 (45.3)		406 (32.8)
Childhood cataract	139 (61.2)	88 (38.8)		227 (18.4)
Infection/inflammation	55 (53.4)	48 (46.6)		103 (8.3)
Orbital/ocular tumor	48 (54.5)	40 (45.5)		88 (7.1)
Congenital anomalies	36 (42.9)	48 (57.1)		84 (6.8)
Childhood glaucoma	51 (67.1)	25 (32.9)		76 (6.1)
Refractive error	30 (46.9)	34 (53.1)		64 (5.2)
Orbital/ocular trauma	38 (73.1)	14 (26.9)		52 (4.2)
Ocular allergies	23 (63.9)	13 (36.1)		36 (2.9)
Congenital nasolacrimal duct obstruction	15 (45.5)	18 (54.5)		33 (2.7)
Ptosis	12 (48.0)	13 (52.0)		25 (2.0)
Cortical visual impairment/blindness	7 (41.2)	10 (58.8)		17 (1.4)
Retinopathy of prematurity	6 (60.0)	4 (40.0)		10 (0.8)
Ectopia lentis	5 (83.3)	1 (16.7)		6 (0.5)
Others	5 (50.0)	5 (50.0)		10 (0.8)
**Total **	**692 (55.9)**		**545 (44.1)**	**1237(100)**

## Discussion

The results of this study provide supporting evidence that pediatric eye disorders vary from one country to another, one region to another, and even one society to another. Therefore, there is a continuing need for further studies to provide up-to-date information on the trend of the problems. Ethiopia lacks robust information about the spectrum and frequency of pediatric eye disorders, although this information is essential for planning and evaluating preventive and curative services for children with ocular morbidity. The spectrum and frequency of pediatric eye disorders in the present study were different from those in previous studies conducted elsewhere in the country [5, 6].

Male children attended pediatric eye clinics more frequently than females in the current study. This finding is consistent with Sethi S et al.’s work that addresses the pattern of common eye diseases in children at the eye department of a teaching hospital [7].

According to studies from around the world, including a few from Ethiopia, older age group children were seen primarily due to chronic recurrent diseases, and the children could report any ocular symptoms to their parents or caregivers [5, 8, 9].

In contrast, this study indicated that a higher frequency of cases was seen in the younger age group of less than 6 years. The early presentation of the children for ophthalmic consultation may be due to the pattern of the eye disorders that were found in this study, which could be detected easily by parents or caregivers.

The present study showed that ocular motility disorders were the most common disorder seen in the study population. A similar type of study performed by Demissie B et al. in southwestern Ethiopia showed ocular surface and eyelid infections as the most common childhood ocular diseases indicating the change in the pattern of pediatric eye disorders within the country according to the difference in the population studied [6].

Cataracts are likely to be the leading cause of blindness in children in West Africa [10]. Childhood cataracts were seen in 18.4% of children in this study. Keeping in mind, the patients were filtered out before they reached the pediatric eye clinic. This finding is comparable with a study performed by CE Gilbert et al. [10].

Infection and inflammation of the eye surfaces and adnexa were observed in this study, representing 8.3% of cases. This pattern of results is consistent with (7.98%) of the Achigbu et al study [11].

The prevalence of tumors in this study was 7.1%, with retinoblastoma accounting for 5.3% and rhabdomyosarcoma accounting for 0.8%. Retinoblastoma is the most common intraocular tumor in childhood. These trends are in line with previous results [12].

Congenital ocular anomalies were statistically associated with and occurring at preschool age, which agrees with other hospital-based studies [13, 14].

We found a much higher value for childhood glaucoma with respect to those reported by the study carried out at Tikrit teaching hospital [9], the Nigeria study [14], and the Pakistan study [7]. This may have occurred because we had referred patients across the country for surgical attention.

Our findings confirm the prevalence of refractive error and are in good agreement with a study performed by Fasih U et al. [12]. As might have been expected, our findings were often contradictory to the notation that hypermetropia was more common during childhood [5]. The difference obtained could be attributable to the age of the children and prolonged use of near vision. In this study, most of the myopic children were of school age.

Although our results defer considerably from those of Onakpoya et al. [13], it can nevertheless be argued that pediatric eye trauma is managed by different duty ophthalmologists assigned for evaluation and repair of any laceration under general anesthesia, and the children will continue their follow-up with the respective ophthalmologist at different ophthalmic clinics in the hospital.

Waddell K M [15] and Okoye O et al [16] noted a higher prevalence of ocular allergies. Our results do not support their observation. In fact, the factors responsible for the difference may be ethnic groups, referral systems and geographical zones.

Congenital nasolacrimal duct obstruction was found in 2.7% of children in our study. A similar report was noted in a study from Pakistan (4.07%) [12].

The results of the current study showed that ptosis had a comparable prevalence to other clinic-based studies [8].

Our findings seem to imply that cortical visual impairments and retinopathy of prematurity are emerging pediatric eye disorders, in contrast with what was previously reported in Ethiopia [5, 6]. The reason could be the increased survival rates of premature and term neonates in the country.

The strengths of this study include the large sample size, the prospective nature of the study and standardization of diagnoses given that they were made by a limited number of pediatric ophthalmologists. However, it was hospital-based and, as such, is not generalizable to the community. Additionally, milder eye conditions are usually treated in primary and secondary care centers, and the profile of diagnoses in the tertiary referral hospital would not reflect them. The diagnoses were mostly clinical.

## Conclusions

In this study, we examined children with ocular disorders presenting for the first time and those with a settled diagnosis for a follow-up visit. After investigating a 1-year eye disease pattern of 1237 children, our statistical analysis concluded that the most common causes of ocular disorders among children attending the tertiary pediatric eye clinic at Menelik II Hospital were ocular motility imbalance, childhood cataract, and infection and inflammation of the eye and adnexa. Early presentation was common, and males were more affected than females. Although previous findings in Ethiopia indicated a variable pattern of eye disorders in a pediatric population, our study followed a greater number of patients than those in studies conducted previously. It is possible that the patterns would vary if determined in different geographic areas or socioeconomic situations and change over time. Future researchers should consider investigating the spectrum of pediatric eye diseases in the general population in different locations and at different times. Regardless, our results point to the need to improve the existing tertiary eye care facilities to reduce the prevalence of needless childhood visual impairment or blindness.

## Data Availability

The datasets used and analyzed during the current study are available from the corresponding author on reasonable request.

## Abbreviations

CSM: Central, Steady and Maintained
N: Number
Yrs: Years
